# Motor Signatures in Digitized Cognitive and Memory Tests Enhances Characterization of Parkinson’s Disease

**DOI:** 10.3390/s22124434

**Published:** 2022-06-11

**Authors:** Jihye Ryu, Elizabeth B. Torres

**Affiliations:** 1 Department of Psychology, Rutgers University, New Brunswick, NJ 08854, USA; jihyeryu@mednet.ucla.edu; 2 Rutgers University Center for Cognitive Science, Computational Biomedicine Imaging and Modeling Center at Computer Science Department, Psychology Department, Rutgers University, Piscataway, NJ 08854, USA

**Keywords:** Parkinson’s disease, micro-movement spikes, digital biomarkers, biometrics, stochastic analysis

## Abstract

Although interest in using wearable sensors to characterize movement disorders is growing, there is a lack of methodology for developing clinically interpretable biomarkers. Such digital biomarkers would provide a more objective diagnosis, capturing finer degrees of motor deficits, while retaining the information of traditional clinical tests. We aim at digitizing traditional tests of cognitive and memory performance to derive motor biometrics of pen-strokes and voice, thereby complementing clinical tests with objective criteria, while enhancing the overall characterization of Parkinson’s disease (PD). 35 participants including patients with PD, healthy young and age-matched controls performed a series of drawing and memory tasks, while their pen movement and voice were digitized. We examined the moment-to-moment variability of time series reflecting the pen speed and voice amplitude. The stochastic signatures of the fluctuations in pen drawing speed and voice amplitude of patients with PD show a higher signal-to-noise ratio compared to those of neurotypical controls. It appears that contact motions of the pen strokes on a tablet evoke sensory feedback for more immediate and predictable control in PD, while voice amplitude loses its neurotypical richness. We offer new standardized data types and analytics to discover the hidden motor aspects within the cognitive and memory clinical assays.

## 1. Introduction

**Figure 1:**
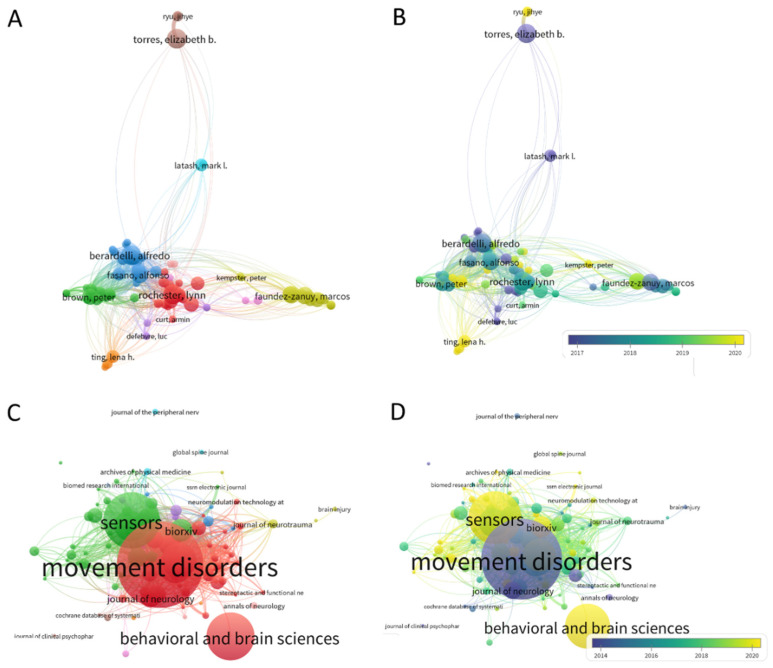
Bibliographic tool VOS Viewer reveals the use of kinematic parameters to characterize symptoms of Parkinson’s Disease. (**A**) Network of authors with main clusters and interconnections according to co-citations. (**B**) Timeline of the work revealing earlier work in 2016 (dark purple) and more recent work in green and yellow tones. (**C**) Density plot of bibliographic couplings revealing the main groups of inter-related vs. unique approaches. (**C**,**D**) Sources publishing the work in (**A**–**C**) reveal the journal of Movement Disorders as the main and earliest source for this type of research, with the journal of Sensors and Behavioral and Brain Sciences as more recent exponents of the work.

The advent of wearable biosensors and their use in research throughout the last decade spurred the use of kinematic parameters to characterize symptoms of Parkinson’s disease (PD) [1,2,3,4,5,6]. For example, a search on app.dimensions.ai using the words kinematic biomarkers and Parkinson’s disease yielded 2990 peer-reviewed papers since 2016, that we can visualize using some features of the VOS Viewer software for bibliographic analyses [7]. Figure 1A,B show clusters of main labs using kinematics for analyses of PD symptoms from 2016 to the present day, and Figure 1C,D show the main peer-reviewed journals publishing this type of research since 2013.

In the past, criteria to assess PD were more reliant on observation and pencil and paper inventories that clinicians have perfected over time, under best-practice standards that include training, certification, and scientific exchange to create reliable tools. Notwithstanding their rigor and ease of use for scalability, relying exclusively on observation misses an opportunity to see beyond the limits of the naked eye. With new off-the-shelf biosensors that have high sampling resolution and research-grade quality, we are now well-positioned to complement the experienced clinical eye with digital data, to produce *interpretable digital biomarkers*. This new concept emerges from digitizing existing clinical tests, e.g., the Universal Parkinson’s Disease Rating Scale (UPDRS) [8] and other cognitive tests such as the Montreal Cognitive Assessment (MOCA) [9] commonly used in clinical settings. Such digital characterization of various aspects of the disorder is easy to do because wearable biosensors are non-obtrusive and can be easily placed on the person’s body, clothing or even collect data streams of motion and voice as the person performs the tasks that are typically administered at clinical settings, or over the internet, e.g., via zoom or using other telemedicine means.

Biosensor’s data offers many advantages, as they increase the level of granularity of the motor phenomena and enable us to learn more about the internal physiological activities of the person’s nervous systems. However, analytical techniques often rely on summary statistics under theoretical assumptions of linearity, normality, and stationarity in the data, when, empirically, the streams of analog data that we collect can change in highly non-linear ways, not be normally distributed and be non-stationary, within the time scale of hours or even minutes. Indeed, we have found that kinematic parameters change nonlinearly [4,6], tend to not distribute normally [10,11] and shift signatures of variability from moment to moment in ways that allow differentiation between patients with PD and controls [5].

For all these reasons, we created a new data type (coined micro-movement spikes, MMS) that permits us to provide an empirically informed characterization of the movement phenomena across different parameter spaces and data stream modalities. This is done by combining information from different data streams (e.g., voice and motion) during typical clinical assessments (Benson complex figure, trail making test, clock drawing) and memory tests. These digitized clinical assays can be very revealing of the personalized signatures and of the cohort’s signatures and trends. Here we employ these new methods and analyze motor signatures from voice and drawing motions during commonly performed cognitive and memory tasks that are part of the clinical assessments used in PD. Our main goal is to bridge digital data with clinical criteria under a common statistical platform that enables the characterization of multiple aspects of PD. Using this approach, we propose to derive clinically interpretable digital biomarkers of PD, while leveraging the scaling capabilities that off-the-shelf sensors offer when registering data on- and/or off-the body.

## 2. Materials and Methods

### 2.1. Participants

A total of 35 participants partook in this study. Among these, 18 participants (age 28–77) were recruited from the Robert Woodrow Johnson Medical Center at Rutgers University or Clinical Trials website (ID NCT03672266). We had 11 patients with PD and 5 age-matched neurotypical participants (see Table A1), and 17 undergraduate students (age 18–35) recruited as young healthy controls from the Rutgers human subject pool system. All participants provided informed consent, which was approved by the Rutgers University Institutional Review Board (#Pro2020000154).

From a series of experiments, we report a subset, namely, the drawing and memory tasks, from which we analyzed the pen motion and voice data respectively. All but two participants performed these in-person, while two of the age-matched controls performed only the memory tasks remotely via Zoom (San Jose, CA, USA).

### 2.2. Experiment Procedure

**Figure 2:**
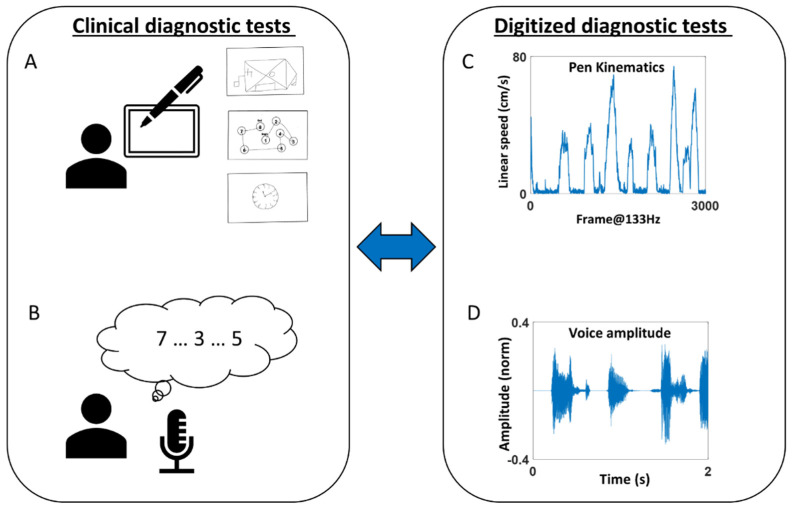
(**A**,**B**) Clinical diagnostic tests of drawing and memory tasks were performed. (**C**,**D**) These were digitized and assessed through kinematic analyses of the pen motion during the drawing task, and audio analyses of the voice during the memory task.

The participant completed two tasks—drawing and memory (Figure 2).

For the drawing task, the participant used a digitizing pen and tablet (Wacom, Japan) to complete seven drawing tasks, which are subtasks of standardized clinical diagnostic tests. Specifically, the tasks were to (1) copy a Benson Complex Figure [12] (denoted as “BCopy” in Figure 2A top), (2) connect 8 circles in numerical order (denoted as “TrailA” in Figure 2A middle), (3) connect 25 circles in numerical order, (4) connect 8 circles composed of 4 numbers and 4 letters in alpha-numerical order (denoted as “TrailB(s)”), (5) connect circles of 13 numbers and 12 letters in alpha-numerical order (denoted as “TrailB”), (6) draw an analog clock and set the time to 10 past 11 (denoted as “Clock” in Figure 2A bottom), and (7) draw the Benson Complex Figure from memory (denoted as “BDelay). Motion from the digital pen was recorded with the software MovAlyzer (Neuroscript; Tempe, AZ, USA), sampling the position of the pen tip at 133 Hz.

For the memory task, the researcher recited a string of numbers (ranging from 2 to 9 digits), and the participant repeated them in the same order for the forward memory task, and in reverse order for the backward memory task [13]. The researcher continued until the participant failed to repeat them in two consecutive trials. The audio of the participant’s voice was recorded with a microphone sampled at 48,000 Hz. For the two participants who completed the task remotely, the voice was recorded from an audio clip provided by Zoom (San Jose, CA, USA), sampled at 44,100 Hz.

### 2.3. Preprocessing Methods

**Figure A1:**
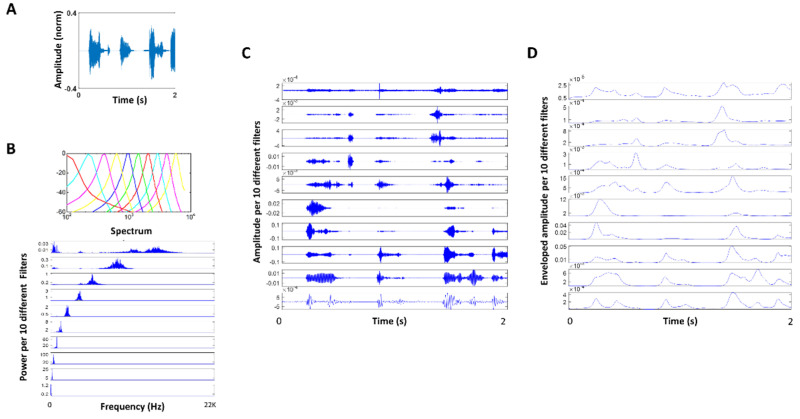
(**A**) Raw audio waveforms during speech. (**B**) Gammatone filter to simulate the basilar membrane response. (**C**) Audio decomposed by Gammatone filter bank. (**D**) Enveloped audio from (**C**).

The audio data collected during the memory tasks were continuously recorded while the researcher and participant alternated saying a string of numbers. We manually removed the researcher’s voice from the audio using Audacity (open-source software; version 2.3.1) and glued the participant’s voice from different trials. Then, the audio was decomposed using the Gammatone filter [14,15] in the MIR toolbox [16] (Appendix A Figure A1A–C). The Gammatone function is defined in the time domain by its impulse response and is known to simulate the response of the basilar membrane [14]. The decomposed audio was then enveloped in the MIR toolbox (Appendix A Figure A1D). This produced 10 different enveloped audio amplitudes, which we examined separately to find which range of band was most informative to characterize PD. Although we found a similar pattern across all bands, we found the 8th band filter (ranging from 4000 to 7000 Hz, centered at 5600 Hz) to be the most informative. For that reason, we present the results based on this filtered enveloped audio data.

Note, two participants’ data were sampled at a different rate (44,100 Hz) from the rest of the participants (48,000 Hz). We analyzed the data by down sampling them to the be at the same rate but did not find much difference in the overall pattern that we would describe in the later section. For that reason, we preserved the audio data at their actual sampling rates in the analysis for everyone.

### 2.4. Data Analysis

#### 2.4.1. Stochasticity of Pen Movement during Drawing Task

**Figure 3:**
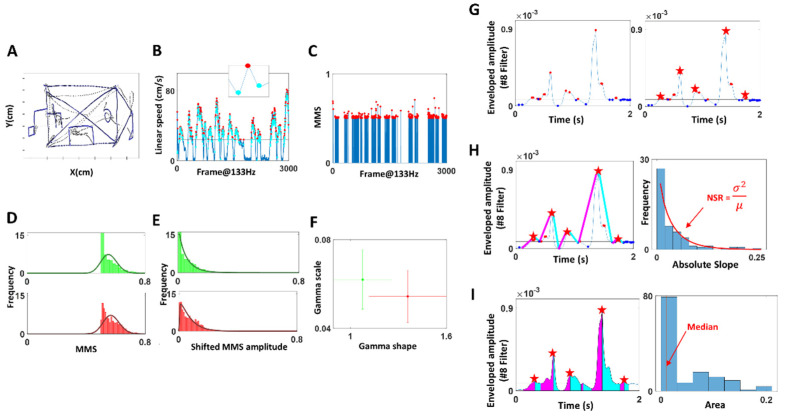
Sample methods of data acquisition for drawing motions (**A**–**F**) and enveloped voice amplitude (**G**–**I**). (**A**) Positional trajectories of drawing a Benson complex figure. (**B**) Speed profiles derived from positional trajectories in A and inset show local maximum (red) surrounded by local minima (cyan). (**C**) MMS spike train created from B, where maxima (red) are normalized to a value between 0 and 1, and non-maxima values are set to 0. (**D**) Distributions of MMS, which do not appear normal. (**E**) MMS values are shifted to the left and are thus well fit by the continuous Gamma family using MLE. (**F**) Gamma shape, scale parameter space to localize each person (with 95% confidence intervals from the empirical MLE process.) Two individuals are plotted as an example. (**G**) Enveloped amplitude of the #8 filter proved useful to characterize PD. Maxima peaks above the median (red) and minima (blue) are identified with a unique selection of maximum in each segment (red star). (**H**) Slopes of attacks and decays are computed and plotted on a histogram with a fitted distribution, and its NSR is computed. (**I**) Areas under the curve for each uniquely defined maximum-segments are plotted on a histogram and its median is identified.

**Figure A2:**
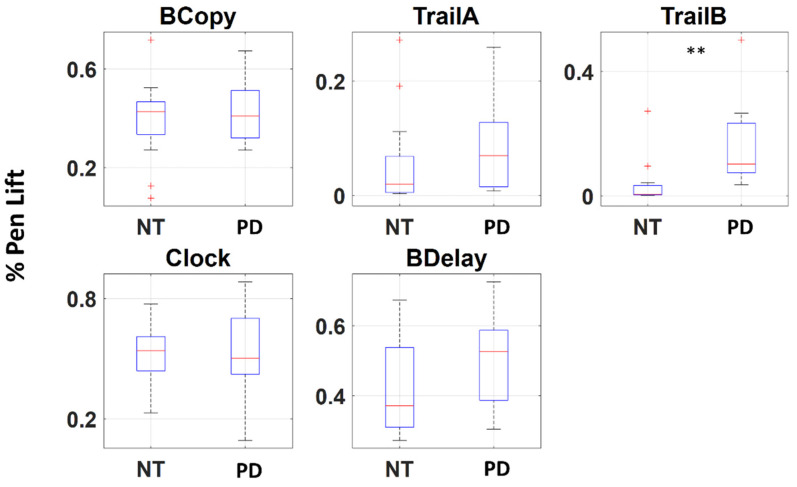
Boxplot of percentage of pen lifts during the entire duration of recording for 5 drawing tasks. ** *p* < 0.01.

The positional trajectory of the pen tip was registered (Figure 3A) with a digitizing pen and tablet (Wacom, Japan), and its linear speed was computed (Figure 3B) by taking the derivative of the positional data (cm). The derivative of position provides a continuous velocity vector field, obtained at a uniform sampling frequency (unit of time.) We then used the Euclidean norm to obtain the scalar quantity of speed, as the distance traveled per unit time (cm/s). Note, linear speed was computed for the entire duration, which includes the times when the pen was lifted from the tablet (see Figure A2 for the percentage of pen lifting). The peaks and minima were extracted from the linear speed time series (denoted in red and cyan, respectively in Figure 3B), which were then converted into a unitless micromovement spike (MMS) data [17,18]. These standardized spike amplitudes were computed using the Equation (1) below:

(1) $$\mathrm{MMS}=\frac{Peak}{Peak+Avrg_{\text{min-to-min}}}$$

Note, we only extracted the MMS values with raw peak amplitudes exceeding the median linear speed (denoted in black horizontal line in Figure 3B), as these high peak amplitudes were most informative to characterize PD motions. The inset in Figure 3B zooms into a speed peak (red) and its surrounding local minima (cyan), which are the elements of computing MMS (Equation (1)). This equation scales out the anatomical differences that would otherwise introduce allometric effects on the analyses [19]. The resulting MMS data is plotted in Figure 3C, where the peak amplitudes are normalized to a value between 0 and 1.

To observe the stochasticity of the pen movement, the MMS (standardized spikes peaks) were plotted on a histogram (Figure 3D), and showed to be concentrated at 0.5, exhibiting a shifted exponential-like distribution shape. To allow a good fit to a family of distributions, the MMS amplitude values were shifted by subtracting 0.5 uniformly (Figure 3E). As a result, the histogram of such shifted MMS was best fit to a Gamma PDF using maximum likelihood estimation (MLE). To characterize PD, the fitted Gamma parameters—shape, scale—were plotted with 95% confidence intervals on a Gamma parameter plane, using different colors (red, green) to represent different members of the cohort (Figure 3F). Eventually, the stochasticity of these pen motions was compared against the clinical scores obtained from the drawings. Specifically, for the task of copying the Benson complex figure (BCopy) and drawing it from memory (BDelay), the drawing scores were based on the accuracy of the drawing from memory (Mscore) and ranged from 1 to 17. The trail-making tests (TrailA, TrailB) were scored based on the time (seconds) that it took to complete the task. Because the sample test of Trail A was short (less than 5 s for most participants), we did not include this in our analysis. Lastly, the clock drawing task (Clock) was scored based on the accuracy of the drawing and ranged from 0 to 3.

Finally, to characterize PD, we took these parameters—Gamma scale (mathematically equivalent to the noise-to-signal ratio NSR which is 1/SNR), Gamma shape, drawing score—and compared the PD against the NT cohort, using a non-parametric Kruskal–Wallis ANOVA test.

#### 2.4.2. Stochasticity of Attack and Decay Phases of Speech

The enveloped audio filtered at 4000–7000 Hz (centered at 5600 Hz) was examined by segmenting the data by attack (upwards) and decay (downwards) phases. First, minima and maxima were extracted, with a criterion that maxima be above the median amplitude’s values, and minima be below the median (Figure 3G, left). This is because we wanted to focus on *meaningful* maxima and minima that were most likely produced by actual speech, and ignore pauses and non-speech segments (e.g., breathing, sighing). Since there were cases where multiple maxima were present between two minima, and because our analytics involved segmenting the attack and decay phases (where the attack phase would start from the local minimum to the subsequent local maximum, and the decay phase start from the local maximum to the subsequent local minimum), it was necessary to have only one maximum between two minima. For that reason, when multiple local maxima were present between two adjacent minima, we chose the maximum (denoted by the red star in Figure 3G, right) as the start and end points of attack and decay phases.

Then, we computed the attack and decay slopes. The attack slope was computed by taking the slope between the local minimum and the subsequent maximum (denoted by the magenta line in Figure 3H), and the decay slope between the maximum and subsequent minimum (denoted by the cyan line). The absolute slopes were then plotted on a histogram and their NSR (i.e., fitted Gamma PDF scale parameter) were compared between PD and other groups. We also computed the area under the curve during the attack and decay phases, by taking the Riemann sum during the two phases as shown in magenta and cyan, respectively, in Figure 3I. Then the stochasticity of these areas was plotted on a histogram and the median values were examined to characterize PD against other groups. Finally, the stochasticity of these attack and decay phases was examined against the clinical scores of the memory task (referred to as “memory score” from here on), which was the average of the longest digits correctly recited during the forward and backward memory tasks. To characterize PD, we performed a series of non-parametric Kruskal–Wallis ANOVA tests on the computed parameters (memory score, NSR, area under the curve) for the PD cohort against the NT cohort.

Note, we tested with other measures (e.g., the SNR of the area under the curve, the median of absolute slopes), but found the SNR or 1/NSR of absolute slopes, and median of the area under the curve to best characterize and differentiate PD from controls. We also tested these parameters separately for the forward and backward memory tasks but did not find much difference between the two. For that reason, we combined the audio from the forward and backward tasks for the analysis.

In our framework, the word standardized refers to four important aspects of our personalized analyses which are a departure from traditional methods currently in use:

(1) The data of interest for analyses are not the absolute value of the original waveform, but rather the relative positive deviation obtained from the empirically estimated mean/median of the set from the individual participant. Since we do not average the absolute amplitude values (or temporal values) under an a priori assumed (theoretical) distribution, our data form is a standardized version of the original. They reflect the rate of change of the values in the time series, relative to the empirical mean.
(2) This standardized deviation from the empirical mean, taken point by point, is now a time series that retains the temporal information of when the original peaks occurred, but is normalized between 0–1 to build a standard metric that is independent of anatomical size differences across the population (Equation (1)), impacting the values of the kinematic parameters of interest (e.g., distance traveled, speed, acceleration, in their linear or angular rotation forms, etc. which are scalar positive values with skewed distributions).
(3) The sampling resolution of the sensors allows us to sample thousands of points/windows of unit time in this standardized space, thus providing enough power to make a statement about the person’s biorhythms under consideration.
(4) The empirical estimation then of the distribution of those values per window and the resulting continuous family of probability distributions representing the overall stochastic process defines a standardized parameter space where we represent the empirically estimated parameter values of the person. It is in this sense that the methods are standardized for personalized statistical inference. Any cluster of points (i.e., probability distributions) representing subtypes, self-emerge in these standardized parameter spaces and can be assessed using appropriate similarity (distance) metrics in probability spaces.)

## 3. Results

### 3.1. Patients with PD Show Higher Signal-to-Noise Ratio (SNR) in Their Pen Motion

**Figure 4:**
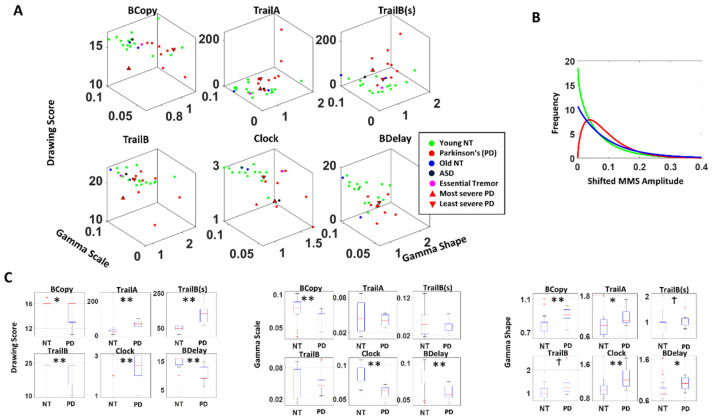
Results from the analyses of drawing tasks. (**A**) Parameter space combining Gamma scale, shape and drawing scores systematically separate the stochastic signatures derived from the drawing motions of young and elderly controls from those of PD patients, with different patterns across different drawing tasks. (**B**) Overall distributions fitting the data also separate PD from controls of different ages, with an exponential fit for controls and skewed Gamma for PD. (**C**) Non-parametric Kruskal–Wallis ANOVA separates PD patients from young NT (denoted as NT) with significance across multiple drawing tests. * *p* < 0.05, ** *p* < 0.01, ^†^ *p* < 0.1.

The fitted Gamma parameters alongside the actual drawing scores were plotted for each participant, for each drawing task in Figure 4A. This figure spans a parameter space amenable to detecting self-emerging patterns across the cohort. We also set landmarks of the most severe case of PD according to the clinical scores (upright triangle) vs. the least severe case (inverted triangle).

**Figure A3:**
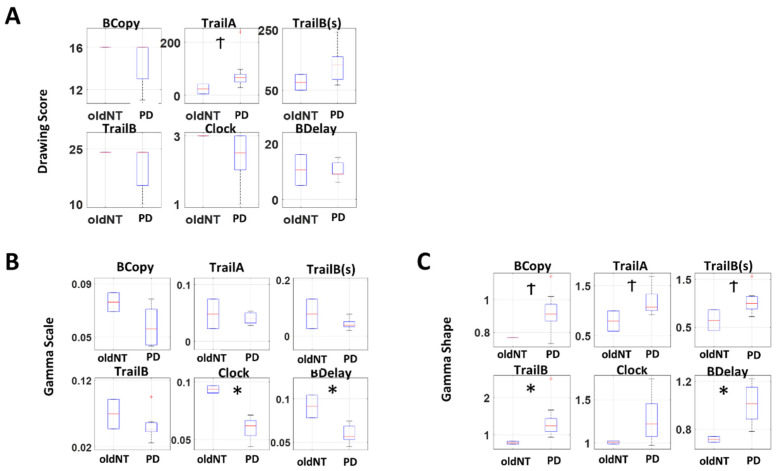
Boxplot of (**A**) Drawing scores and (**B**,**C**) Gamma parameters between age-matched neurotypical (oldNT) and Parkinson’s patient. * *p* < 0.05; ^†^ *p* < 0.1.

For all drawings, patients with PD showed lower drawing scores than the young healthy cohort (NT), (Figure 4C left, Table 1). When we examine the stochasticity of pen motion, PD tends to have a lower scale (i.e., lower NSR, higher SNR) and higher shape parameter than NT (Figure 4 middle, right, Table 1). The single participant with ASD and the one patient with Essential Tremor did not show noticeable differences in their stochasticity, compared to NT. The separation between PD and NT is most pronounced during the clock and the Benson complex figure task (both BCopy and BDelay), which involves drawing on a blank sheet of paper, which allows more freedom in motion. These tasks contrast with others, where participants are given a paper with some form of figures already printed on it (e.g., circles with letters and/or numbers), and thus have less freedom in their pen motion. We also analyzed this separately between older NT and the PD, and still found similar patterns with a reduced difference in the stochasticity as shown in Figure A3, thus confirming that the observed pattern between PD and NT is not solely due to age.

When we examined the PDF, the typical patient with PD tends to have a flatter distribution with less skewness than NT, as shown in Figure 4B. This alludes to the nature of contact control (control while body is in contact with an object) among individuals with PD, where they tend to exhibit lower NSR (i.e., higher SNR) in their hand motion stochasticity, especially in cases where there is less restrictions to their movement (as in the clock and Benson figure tasks). This can be a useful addition to the current drawing diagnostic tests, because certain drawing test scores (particularly the clock and Benson figure copy) tend to be too coarse to differentiate participants, as most end up reaching the ceiling score.

### 3.2. Patients with PD Show Lower Change and Higher SNR in Voice Amplitude

**Figure 5:**
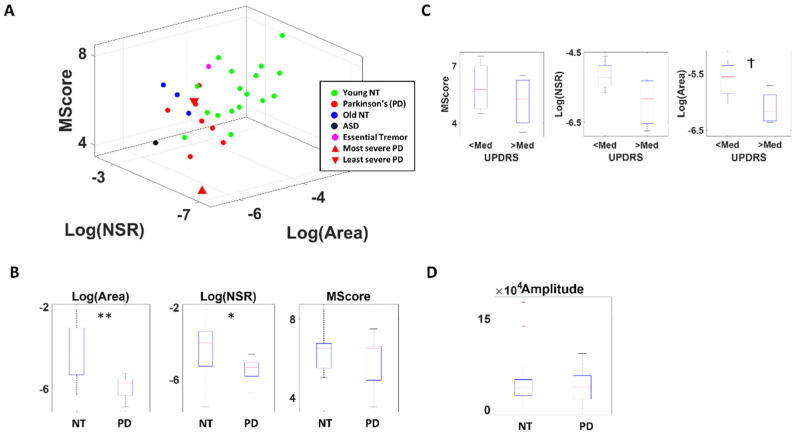
Results from audio analyses from the memory task. (**A**) Parameter space localizing each person according to the stochastic features and clinical memory score and provides interpretable biomarker of voice, separating young controls from PD and other disorders. Axes are log Gamma scale (NSR), log of the area under the enveloped curve (Area), and memory score (MScore). (**B**) Non-parametric Kruskal–Wallis separates participants with PD from young controls (NT) at significance level for Area and NSR. (**C**) The three parameters from (**A**) differentiate between PD with high and low severity. (**D**) The voice amplitudes between NT and PD are similar. * *p* < 0.05, ** *p* < 0.01, ^†^ *p* < 0.1.

Based on the enveloped audio filtered at 4000–7000 Hz, the NSR of the distribution of absolute attack and decay slopes, and the median area under the curve during the attack and decay phases, alongside the actual memory scores obtained during the memory task are plotted for each participant in Figure 5A.

**Figure A4:**
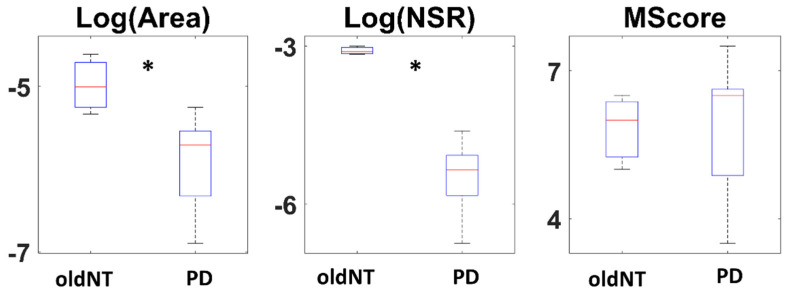
Boxplot logarithm of area, of logarithm of slope NSR, and of memory score between age-matched neurotypical (oldNT) and Parkinson’s patient (PD). * *p* < 0.05.

The memory score itself does not show a statistical difference between PD and NT, although PD tends to show lower scores (χ(1.27) = 0.31, *p* = 0.58). On the other hand, there is a clear separation between the two groups when comparing the area under the curve (χ(1.27) = 9.98, *p* < 0.01) and the NSR of absolute slope distribution (χ(1.27) = 4.70, *p* = 0.03) (Figure 5B). Specifically, PD tend to show a smaller area under the curve and lower NSR (i.e., higher SNR) in their distribution, and this pattern is maintained when comparing PD against their older NT counterparts (for the area under curve, χ(1.10) = 5.34, *p* = 0.02; for NSR of slope distribution χ(1.10) = 6.23, *p* = 0.01) confirming that this difference is not due to age (Figure A4).

**Figure A5:**
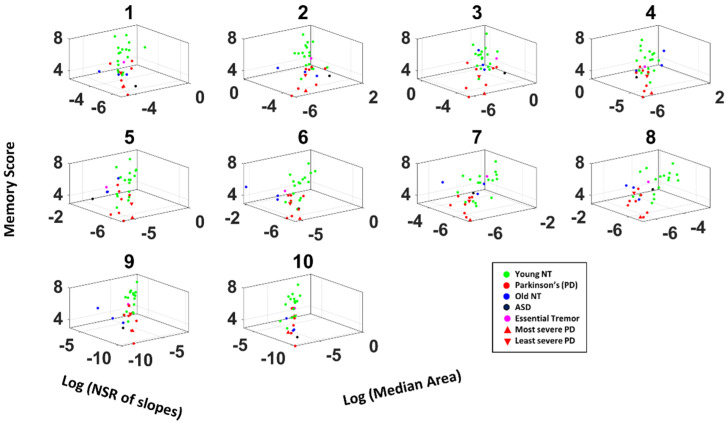
3D plot of memory score, logarithm of slope NSR, and logarithm of median area for all participants, based on differently filtered voice data (#1–10).

When we examine this question only among the PD group and compare those with higher severity (with UPDRS above the median) against those with less severity (with UPDRS below the median) as shown in Figure 5C, we find a similar pattern, suggesting that this measure not only characterizes PD but also reflect the severity of the disorder. To exclude such separation to be due to difference in voice volume, we also compared the voice amplitude between PD and NT and found the two groups to have a similar range of volume in their voices (Figure 5D). We also tested such comparison across different audio bands, and found similar patterns, but most pronounced at frequency 4000–7000 Hz (Figure A5), which is what we show in Figure 5.

Overall, these findings imply that the variability in attack and decay phases of the voice from patients with PD are stochastically different, such that changes in voice amplitude (particularly within the 4000–7000 Hz range) is smaller and less variable than the NT, thus exhibiting higher SNR. These characteristics may be useful additions to cognitive tests, as accompanying deficits in the neuromotor aspects of PD.

## 4. Discussion

In this study, we demonstrate methods to characterize the stochastic signatures of the drawing speed from a hand-held digital pen, and the vocal waveforms of patients with PD. In this cohort, we find a higher signal-to-noise ratio (mean speed/speed variance) in their pen motions and lower richness in the voice amplitude, compared to neurotypical individuals. This work extends the fast-growing body of knowledge involving kinematic analyses of motion patterns from patients with PD and controls across different age groups. Furthermore, we add new analytical methods and a unifying data type for voice analyses, to the vast literature tracking vocal characteristics in PD [20,21,22], including studies that use smart phones to track kinematic features of voice [23], daily motions [5], and those connecting voice changes with gait patterns [24].

We combined data registered from drawing motion trajectories of a digital pen on a digitizing tablet and the participant’s voice registered with commercially available mics, to offer new methods amenable to use in the lab, clinic or remotely over zoom, as the person undergoes traditional clinical evaluations. Remaining unobtrusive, these biosensors digitized and typically administered tests such as parts of the MOCA, assessing memory and cognitive status of the person, and took advantage of the (hidden) motor components of these tests. Using a new data type, the MMS and a combination of non-parametric statistical analyses and data-driven stochastic analyses, we were then able to demonstrate clear separations between patients with PD and controls.

We found that in the drawing tasks, patients with PD manifest lower values of the Gamma scale and higher values of the Gamma shape derived from the MMS of their drawing speed. Their motions are far more controlled and predictable than those of neurotypical young and older participants. The latter are well characterized by fluctuations in speed that distribute exponentially, in contrast to those of PD who had speed fluctuations distributed in skewed to symmetric shape ranges.

This result contrasts with the signatures of pointing motions to visual targets, whereby lower SNR and exponentially distributed fluctuations prevail in PD, denoting higher speed variance than mean speed and more random temporal patterns relative to controls [5,6,25]. Consistent with previous work, this excess variability in pointing kinematics had been reported in earlier studies that used traditional statistical analyses, across larger cohorts of patients with PD [26,27,28]. We attribute these differences to the pen’s contact with the tablet, providing continuous feedback to the motor performance. In PD, motor control is already thought to be under deliberate supervision of the motor systems [29], partly accounting for the bradykinetic motions known to characterize their motor performance. Unlike in pointing motions, where no contact surface provides feedback and the feedback is rather kinesthetic from the fluctuations of the motion trajectories themselves, here we can appreciate this overt supervision with significantly lower speeds and highly controlled fluctuations of the drawing traces, in relation to controls. This result is important because, in addition to offering new ways to use e.g., the MOCA tests to assess motor components, it suggests a new possible target for treatment. Namely, a possible new way to directly provide feedback to the motor systems is through contact motions, that through this pressure-based reafferent channel could aim at increasing the SNR of their fluctuations in traces’ speed, to levels comparable to those of controls.

It is noteworthy that fitting the Gamma PDF allows us to examine a wide range of probability distributions, from exponential to Gaussian [11,17,30], and to interpret the stochastic results with mathematical meaning. Specifically, the Gamma shape parameter ranges from a value of 1 to above 100. The special case of shape = 1 corresponds to the memoryless exponential distribution, whereby immediate future events are equally probable, and no predictive pattern drives the system. This is the case when information is random and exists in “*the here and now*”. As the shape value increases, the distribution shifts from skewed (with heavy tails) to symmetric, highly predictive (Gaussian) patterns. Empirically, we have previously seen high randomness in the motor stochastic signatures of patients with PD [5] and interpreted that the type of kinesthetic (reafferent) feedback that such signatures reveal is less than ideal to predict the consequences of (efferent) action signals, and to compensate for sensory-motor transduction and transmission delays in the system [6,17,31].

The other parameter, the Gamma scale, is equivalent to the noise-to-signal ratio (NSR) (i.e., variance over mean, because given *a* as the Gamma shape and *b* as the Gamma scale, the Gamma mean is a⋅b and the Gamma variance is $a\cdot b^{2}$ such that their noise to signal ratio $\frac{\Gamma \sigma }{\Gamma \mu }=\frac{a\cdot b^{2}}{a\cdot b}=b$, the Gamma scale). Empirical work relating to the log-log parameters shows an inverse relationship between the log NSR (log *b*) and the log shape (log *a*). Gaussian regimes correspond to high SNR (low NSR), and we empirically find these in healthy controls, yielding highly interpretable value to our approach. Indeed, prior studies have demonstrated the NSR to be high among patient populations (e.g., ASD [32], PD [6], schizophrenia [33]) using their kinematics during naturalistic motions and it be susceptible to change with higher motor intent (e.g., pointing [34]).

Using these methods on the voice data, we also found ways to separate patients with PD from controls and demonstrated the differences in parameter spaces for characterizing PD. These methods of data acquisition and analyses are amenable to use with Zoom (San Jose, CA, USA), for remote research and add to our arsenal of instruments that the recent pandemic generated to enable continuity of our research programs using off-the-shelf means.

Nevertheless, the study is not without limitations. Although patients with PD were expected to have taken their regular dosage of medication, they were not explicitly asked about their recent medications. For that reason, we are unsure how/whether medication would have affected the results. Furthermore, the relatively small sample size may not allow the cohort results to have sufficient statistical power to infer population results. However, we point out that each data point from a single participant is a summary of a large, personalized dataset (in the magnitudes of >1000 data measurements.) In this sense, the study yields sufficiently high confidence intervals for our empirical distributional estimation. It is in this personalized sense that our methods yield high statistical power.

The methods presented in this work are personalized, as they reflect the person’s stochastic signatures, uniquely localizing every one of the cohorts on a parameter space. Furthermore, interpretation of the data from the group is possible as clusters self-emerge within the parameter space, often automatically separating the patients from the neurotypical controls. As the data are standardized and possible allometric effects scaled out, the MMS derived from the moment-to-moment fluctuations in signal amplitude (e.g., speed or other derived parameters such as area under the curve), we can add other patient populations with motor issues, so as to begin the process of automatically stratifying the heterogeneous spectrum of disorders and identifying which parameters serve to maximally separate diverse disorders of the nervous systems, using motoric components hidden in these minute fluctuations that we tend to throw away as superfluous, gross data. Here we made good use of these standardized fluctuations, offering new ways to leverage existing cognitive and memory inventories broadly used by the field of clinical and basic research.

In summary, we offer new standardized data types and analytics to help advance the clinical and applied research of Parkinson’s disease.

## Figures and Tables

**Table 1:** Kruskal–Wallis Test on Parameter Comparison during drawing task.

Parameter	Drawing	yNT ^1^ vs. PD		oldNT ^2^ vs. PD	
		Chi (1.25)	*p*-Value	Chi (1.10)	*p*-Value
Drawing Score	BCopy	5.80	0.02 *	0.72	0.40
	Trail A	14.95	0.00 **	2.95	0.09
	Trail B(s)	16.54	0.00 **	2.26	0.13
	Trail B	7.63	0.01 **	1.05	0.31
	Clock	6.95	0.01 **	1.50	0.22
	BDelay	7.77	0.01 **	0.00	1.00
Gamma shape	BCopy	7.08	0.01 **	2.95	0.09
	Trail A	4.66	0.03 *	2.95	0.09
	Trail B(s)	3.45	0.06	2.95	0.09
	Trail B	3.64	0.06	4.62	0.03 *
	Clock	7.80	0.01 **	2.26	0.13
	BDelay	4.31	0.04 *	4.50	0.03 *
Gamma scale	BCopy	6.82	0.01 **	2.26	0.13
	Trail A	0.00	1.00	0.00	1.00
	Trail B(s)	0.06	0.80	0.05	0.83
	Trail B	0.57	0.45	1.15	0.28
	Clock	15.21	0.00 **	4.62	0.03 *
	BDelay	8.63	0.00 **	4.50	0.03 *

^1^ yNT: Young neurotypical; ^2^ oldNT: age-matched neurotypical; * *p* < 0.05; ** *p* < 0.01.

## Data Availability

Data available on request to the corresponding author, due to privacy restrictions.

## Appendix A

**Table A1:** Demographics of patient and age-matched neurotypical participants.

Participant ^1^	Gender	Age	UPDRS	Hoehn and Yahr Scale	Medication (Motor/Non-Motor) ^2^	Pen Data	Voice Data
PD 1	M	51–60	6	2.5	Yes/Yes	Yes	Yes
PD 2	F	61–70	44	4	Yes/Yes	Yes	Yes
PD 3	M	61–70	24.5	2.5	Yes/Yes	Yes	Yes
PD 4	M	61–70	19	3	Yes/Yes	Yes	Yes
PD 5	M	51–60	29	3	Yes/Yes	Yes	No
PD 6	F	61–70	-	3	Yes/No	Yes	Yes
PD 7	M	61–70	16	2	Yes/Yes	Yes	Yes
PD 8	M	71–80	21	3	Yes/No	Yes	Yes
PD 9	F	71–80	27	3	Yes/Yes	Yes	No
PD 10	M	41–50	-	2	Yes/Yes	Yes	Yes
PD 11	M	61–70	20	-	-	No	Yes
ASD 1	F	21–30	-	-	-	No	Yes
ET 1	M	31–40	-	-	-	Yes	Yes
Old NT 1	F	61–70	-	-	-	Yes	No
Old NT 2	M	61–70	-	-	-	Yes	No
Old NT 3	F	51–60	-	-	-	No	Yes
Old NT 4	F	71–80	-	-	-	No	Yes
Old NT 5	M	71–80	-	-	-	No	Yes

^1^ Participant Abbreviation; PD: Parkinson’s disease; ASD: Autism spectrum disorder; ET: Essential tremor; Old NT: age matched neurotypical control; ^2^ Intake of medication for motor issues/medication for non-motor issues that are known to affect motor performance.
